# ALDH3A1 Plays a Functional Role in Maintenance of Corneal Epithelial Homeostasis

**DOI:** 10.1371/journal.pone.0146433

**Published:** 2016-01-11

**Authors:** Vindhya Koppaka, Ying Chen, Gaurav Mehta, David J. Orlicky, David C. Thompson, James V. Jester, Vasilis Vasiliou

**Affiliations:** 1 Department of Pharmaceutical Sciences, Skaggs School of Pharmacy and Pharmaceutical Sciences, University of Colorado Anschutz Medical Campus, Aurora, Colorado, United States of America; 2 Department of Environmental Health Sciences, Yale School of Public Health, New Haven, Connecticut, United States of America; 3 Department of Pathology, University of Colorado Anschutz Medical Campus, Aurora, Colorado, United States of America; 4 Department of Clinical Pharmacology, University of Colorado Anschutz Medical Campus, Aurora, Colorado, United States of America; 5 Department of Ophthalmology, Gavin Herbert Eye Institute, University of California Irvine, Irvine, California, United States of America; Cedars-Sinai Medical Center; UCLA School of Medicine, UNITED STATES

## Abstract

Aldehyde dehydrogenase 1A1 (ALDH1A1) and ALDH3A1 are corneal crystallins. They protect inner ocular tissues from ultraviolet radiation (UVR)-induced oxidative damage through catalytic and non-catalytic mechanisms. Additionally, ALDH3A1 has been postulated to play a regulatory role in the corneal epithelium based on several studies that report an inverse association between ALDH3A1 expression and corneal cell proliferation. The underlying molecular mechanisms and the physiological significance of such association remain poorly understood. In the current study, we established Tet-On human corneal epithelial cell (hTCEpi) lines, which express tetracycline-inducible wild-type (wt) or catalytically-inactive (mu) ALDH3A1. Utilizing this cellular model system, we confirmed that human ALDH3A1 decreases corneal cell proliferation; importantly, this effect appears to be partially mediated by its enzymatic activity. Mechanistically, wt-ALDH3A1, but not mu-ALDH3A1, promotes sequestering of tumor suppressor p53 in the nucleus. In the mouse cornea, however, augmented cell proliferation is noted only in *Aldh1a1*^*-/-*^*/3a1*^*-/-*^ double knockout (DKO) mice, indicating *in vivo* the anti-proliferation effect of ALDH3A1 can be rescued by the presence of ALDH1A1. Interestingly, the hyper-proliferative epithelium of the DKO corneas display nearly complete loss of p53 expression, implying that p53 may be involved in ALDH3A1/1A1-mediated effect. In hTCEpi cells grown in high calcium concentration, mRNA levels of a panel of corneal differentiation markers were altered by ALDH3A1 expression and modulated by its enzyme activity. In conclusion, we show for the first time that: (i) ALDH3A1 decreases corneal epithelial proliferation through both non-enzymatic and enzymatic properties; (ii) ALDH1A1 contributes to the regulation of corneal cellular proliferation *in vivo*; and (iii) ALDH3A1 modulates corneal epithelial differentiation. Collectively, our studies indicate a functional role of ALDH3A1 in the maintenance of corneal epithelial homeostasis by simultaneously modulating proliferation and differentiation through both enzymatic and non-enzymatic mechanisms.

## Introduction

As the anterior most segment of the mammalian eye, the cornea represents the first line of defense against external insults. The structure and function of the cornea is integral in imparting deturgescence and transparency for normal vision. The corneal epithelium exists in a constant state of turnover through the lifetime of a mammal. The processes of cell proliferation in the basal layers, differentiation in the superficial layers, post-mitotic terminal differentiation and cell death at the surface are all critically balanced to achieve corneal epithelial homeostasis and transparency [1].

The corneal proteome as proposed by the "refracton" hypothesis [2, 3] shares many similarities with the lens proteome in expressing high concentrations (5–40%) of water-soluble enzymes called 'crystallins' in a taxon-specific manner, which are essential in preserving ocular transparency. In other tissues however, the same gene encoding the lens crystallin specifies either a stress protein or a metabolic enzyme with no associated refractive function. Termed as gene sharing, it has been proposed that this phenomenon of a distinct protein with multiple functions may also be extended to the transparent cornea due to the abundant expression of similar water-soluble enzymes/crystallins in the corneal epithelium [4, 5]. The aldehyde dehydrogenases ALDH1A1 and ALDH3A1, analogous to lens crystallins, are abundantly expressed in the cornea and shown to play a vital structural role in preserving corneal transparency as well as a metabolic role in protection against ultra-violet radiation damage [6, 7]. It has further been discovered that both catalytic and non-catalytic activities of these proteins contribute to their metabolic and structural functions in the cornea. Catalytically, ALDH1A1 and ALDH3A1 protect the cornea by (i) eliminating reactive aldehydes, such as 4-hydroxy-nonenal (4-HNE) and malondialdehyde (MDA), arising from UVR-initiated reactive oxygen species and lipid peroxidation [8], (ii) scavenging free radicals through the cysteine residues on the surface of ALDH3A1 and (iii) producing the antioxidant NADPH [9]. Non-catalytically, the enzyme supports corneal transparency similar to lens crystallins, through chaperone activity [10].

A novel regulatory role of ALDH3A1 was first identified when the proliferation rate of human corneal epithelial (HCE) cells was observed to decrease with expression of ALDH3A1. These cells displayed increased population doubling time and decreased plating efficiency and DNA synthesis relative to cells not expressing ALDH3A1 protein [11]. Cyclins A, B and E, E2F and p21 were down-regulated, retinoblastoma protein was dephosphorylated and cyclin-dependent kinase activity was also reduced [11]. Interestingly, transfected ALDH3A1 translocated to the nucleus, suggesting a role for this protein in mitotic regulation [8, 11, 12]. Consistent with this proposed regulatory role, primary human corneal epithelial cells grown in culture progressively lose ALDH3A1 expression. Similarly, in corneal wound healing models *in vivo*, ALDH3A1 expression is down-regulated immediately after injury, coinciding with the period of active proliferation of basal cells around the site of injury [13]. In this study, we explored the anti-proliferative effect of ALDH3A1 in the human telomerase-immortalized corneal epithelial (hTCEpi) cells and knockout mouse models of ALDH3A1 and/or ALDH1A1. Our results confirmed the growth retardation effects of ALDH3A1 and suggested a role of ALDH3A1 in modifying differentiation programs in the corneal epithelium and importantly the involvement of both catalytic and non-catalytic properties of ALDH3A1.

## Materials and Methods

### Cell culture

The parental human telomerase-immortalized corneal epithelial (hTCEpi) cell line has been characterized previously [14]. We obtained this cell line from Dr. Jester’s laboratory at the University of California Irvine. hTCEpi cells were routinely maintained in serum-free keratinocyte growth media (KSFM) (with 0.15 mM Ca^2+^) supplemented with bovine pituitary extract (BPE), insulin, hydrocortisone, penicillin, streptomycin, amphotericin B and human epidermal growth factor (hEGF) (KGM™-CD from Lonza Clonetics, Walkersville, MD). Unless specified elsewhere, cells were passaged every 4 days using 0.25% trypsin-EDTA and 0.25 mg/ml soybean trypsin inhibitor (Invitrogen, Carlsbad, CA) and cultured in T75 tissue culture flasks (Falcon Labware; BD Biosciences, Bedford, MA) incubated at 37°C in 5% CO_2_.

### Construction of lentiviral expression constructs

The full-length cDNA of human wild-type ALDH3A1 was amplified by PCR and subcloned into an entry plasmid pENTR-3A1wt using the pENTR™ Directional TOPO^®^ Cloning Kit (Invitrogen, CA) according to the manufacturer’s protocol. Another entry plasmid, pENTR-3A1mu, harboring a C244A mutation (TGT→GCT) was generated using the Site-directed Mutagenesis kit (Invitrogen, CA) according to the manufacturer’s protocol. The two entry plasmids were then used to generate the lentiviral expression constructs pLEnti4/TO/V5-3A1wt (pLenti-3A1wt) and pLEnti4/TO/V5-3A1mu (pLenti-3A1mu) *via* homologous recombination between the entry clone and the pLenti4/TO/V5-DEST vector according to the manufacturer’s protocol. Coding sequences in the final expression constructs were verified by DNA sequencing.

### Production of lentiviral particles

293FT cells (Invitrogen, CA) were seeded at 80% confluence in a 60 mm dish (Falcon Labware; BD Biosciences, Bedford, MA) and allowed to attach overnight. On the day of transfection, the cells were at 95% confluency and were then incubated for 8 hr with the transfection mixture containing 2 μg pLenti plasmids, 6 μg ViraPower Packaging Mix™ and 20 μl Lipofectamine 2000 reagent. The pLenti plasmids included pLenti6/TR (the plasmid expressing the tetracycline repressor (TR)), pLenti-3A1wt, pLenti-3A1mu or pLenti4/TO/V5-DEST (empty vector control). During the incubation, the cells were kept in 3% Opti MEM medium (Invitrogen, CA). At the end of the incubation period, the medium was replaced by regular growth medium (high glucose DMEM (4.5 g/l) / 10% FBS). Seventy-two hrs later the medium (5 ml) containing the lentivirus particles was collected and centrifuged at 1000xg to remove cell debris. The supernatant was filtered and stored at -80°C for future use.

### Generation of stably transduced hTCEpi cell lines expressing tetracycline-inducible ALDH3A1

hTCEpi cells were first transduced with viral particles of the pLenti6/TR clone and selected in growth medium containing 3 μg/ml blasticidin for 3–4 wk. Surviving single-cell colonies were examined for tetracyclin repressor (TR) expression by Western blot. The pLenti6/TR colony expressing the highest level of TR was subsequently transduced with viral particles of the pLenti-3A1wt, pLenti-3A1mu or pLenti4/TO/V5-DEST clones. Cells were then selected in growth medium containing 3 μg/ml blasticidin and 100 μg/ml zeocin for 3–4 wk. Surviving single-cell colonies were examined for ALDH3A1 expression following tetracycline (TET; 0.01–1.0 mg/L) treatment. The three cell lines were designated as hTCEpi-TR-3A1wt (tetracycline-regulated wild-type ALDH3A1-expressing cells), hTCEpi-TR-3A1mu (tetracycline-regulated mutant (catalytically-inactive) ALDH3A1-expressing cells), and hTCEpi-TR-Lenti (mock control cells).

### ALDH3A1 enzymatic activity assay

Determinations of ALDH activity were carried out using a spectrophotometer (Beckman Instruments, Fullerton, CA) by monitoring NADPH production at 340 nm as previously described [12]. Enzyme activities are presented as nmoles of NADPH produced/min/mg of total protein. Results are reported as mean ± S.E of three biological replicates.

### Cell proliferation assay

Following 4.5 wk culturing of hTCEpi cells, cumulative population doublings (CPD) and population doubling times (PDT) were determined as follows. Cells were seeded in 100 mm culture dishes (2 x 10^5^ cells/dish) and treated with or without tetracycline (1 μg/ml, Invitrogen) in 10 ml of culture media (control). After 4 d, cells were trypsinized and viable cells were counted using trypan blue exclusion on a hemocytometer. Consecutively, 2 x 10^5^ cells were transferred to a new dish and this process repeated for a total of 8 passages, i.e., for 32 d until treated cells showed decreasing cell numbers and could not be plated further at the same density. CPD was calculated as: CPD = ln [N_E_/N_B_] / ln 2 + CPD_o_ (N_E_: end cell count; N_B_: cell count in the beginning; CPD: CPD_o_ at previous passage). PDT at each passage was calculated as PDT = ln (2)/Growth rate. Growth Rate = number of doublings that occur per unit of time, i.e., the slope of the cumulative population doublings curve. Results are reported as mean ± S.E of three biological replicates for each data point.

### Animals

Generation of *Aldh1a1*^*-/-*^ and *Aldh3a1*^*-/-*^ single knock-out (KO) and *Aldh1a1*^*-/-*^*/Aldh3a1*^*-/-*^ double knock-out (DKO) mice has been previously described [15–17]. All three lines have been re-derived into the C57BL/6J background. C57BL/6J wild-type (WT) mice were purchased from the Jackson’s Laboratory. All animal experiments were approved by and conducted in compliance with Institutional Animal Care and Use Committee of the University of Colorado Anschutz Medical Campus (approved protocol #: B33411(01)1D). Mice were maintained in a temperature-controlled room (21–22°C) on a 12 hr light/dark cycle and supplied with food and water *ad libitum*. Six-10 mice from each genetic strain (3–6 mo of age) were euthanized by carbon dioxide followed by cervical dislocation. All efforts were made to minimize suffering. Whole eyes were removed for use immediately in protein extraction or fixed in paraformaldehyde and paraffin-embedded for immunohistochemical analysis.

### 5-bromo-2'-deoxyuridine (BrDU) labeling and immunohistochemistry

For BrDU labeling of mouse corneal epithelial cells, a dose of 100 mg/kg BrDU in Dulbecco's phosphate-buffered saline (DPBS) was administered (i.p.) to WT and DKO mice. Two hr thereafter, mice were euthanized by carbon dioxide inhalation. The eyes were immediately removed, fixed in 4% paraformaldehyde and embedded in paraffin according to standard procedures. Sagital sections (5 μm) were then deparaffinized, rehydrated and blocked for endogenous peroxidase activity by incubation in 1% H_2_O_2_. For antigen retrieval, slides were boiled in 10 mM sodium citrate containing 0.05% Tween-20 (pH 6.0) for 15 min and subsequently incubated with pre-activated RNase A (100 μg/ml in 50mM Tris, pH 7.5, Roche, Indianapolis, IN) for 30 min. The RNA-free DNA was then denatured in 4N HCL at room temperature for 5 min. The pH was then neutralized with 50 mM Tris (pH 7.5) and the tissue section was blocked with Tris-NaCl blocking buffer (TNB, 0.10 M Tris.HCl, 0.15 M NaCl, 0.5% blocking reagent (Roche, Indianapolis, IN)) solution in a humidified chamber. For p53 and Ki-67 staining, the RNAse treatment and DNA denaturation steps above were omitted. Primary antibodies (Anti-BrDU antibody 1:100 BD Biosciences, San Jose, CA; Anti-Ki-67, 1:100; Anti-p53 (FL-393) 1:50, SantaCruz, CA) were diluted in TNB, placed on the section and incubated at 4°C overnight. Following washing in DPBS, the tissue section was incubated for 1 hr with horseradish peroxidase (HRP)-conjugated secondary antibody (1:500) after which the signal was amplified by incubation in biotin-tyramide solution (Perkin Elmer, Waltham, MA) for 5 min. Slides were washed and incubated in streptavidin-conjugated HRP for 30 min at RT (25°C). 3-Amino-9-ethylcarbazole (AEC) (BD Biosciences, Bedford, MA) was applied to the section to facilitate visualization of the labeled protein. Immunohistochemical images were captured on a digital camera (Nikon DS-Fi1-L2) fitted to a microscope (Nikon Eclipse E200). Corneal sections from the same animal were hematoxylin and eosin (H&E) stained according to standard procedure.

The corneoscleral junction was used to define the limit of the cornea from limbus to limbus. The mouse cornea was circular and regions from central and peripheral cornea were visualized at 10X magnification. A 1280 X 960 pixel frame was chosen on central and peripheral cornea (200X magnification) of each mouse to count both BrDU-positive cells and the total number of cells from corresponding H&E-stained sections. The number of cells staining positive for BrDU (N_B_) was expressed as a percentage of the total number of cells stained by hematoxylin (N_H_) as N_B_/N_H_ x 100. Results are presented as mean ± S.E. (N = 3). Total Ki-67 positive nuclei in the entire length of the corneal epithelium of each mouse sagittal eye section were counted at 10X magnification. Results are presented as mean ± S.E. (N = 3).

### High calcium-induced differentiation in hTCEpi cells

hTCEpi cells were incubated in vehicle (KSFM growth media) with 0.15 mM calcium for 4 d (i.e., until cells reached 80% confluency) or in vehicle with 1.15 mM calcium for 7 d (i.e., at which time cells were post-confluent and differentiated). The media was changed every other day in both conditions. For relative mRNA quantification: hTCEpi-TR-Lenti, hTCEpi-TR-3A1wt or hCTEpi-TR-3A1mu cells were seeded at 3 x 10^4^ cells/ml in 6-well plates and incubated for 4d in medium containing (i) low (0.15 mM) calcium or (ii) low (0.15 mM) calcium + TET (1 mg/L) to induce ALDH3A1 expression. Cells were then harvested for total RNA. The same experiment was conducted in cells incubated in medium containing (i) high (1.15 mM) calcium or (ii) high (1.15 mM) calcium + TET (1 mg/L) for 7 d after which total RNA was extracted from cells. The relative mRNA levels represent the amount of mRNA expression normalized to GAPDH.

### Real time–quantitative PCR (Q-PCR)

Total RNA was isolated (RNeasy kit; Qiagen, Valencia, CA) from cultured cells and quantified using Nanodrop 2000 UV/Vis spectrophotometer (Thermo fisher, Waltham, MA). Using 1 μg of total RNA, reverse transcription was carried out using Maxima H minus First strand cDNA synthesis kit (Thermo fisher, Waltham, MA) with Oligo dT primers. Q-PCR was performed with a thermal cycler (IQ5, Biorad, Hercules, CA) using Power SYBR® Green PCR Master Mix (Life technologies, NY) and Primetime qPCR assays (IDT, San Jose, CA). PCR primer sets for respective genes are presented in S1 Table. The final concentration of the primers in the PCR reaction was 500 nM. ALDH3A1 mRNA abundance was quantified by fitting Q-PCR data to a standard curve of copy numbers generated from an *ALDH3A1* cDNA construct as previously described [18]. For other genes, relative mRNA expression levels in fold change are reported after normalization to the housekeeping gene GAPDH using the comparative C_T_ method [19].

### Preparation of cell lysates

For ALDH3A1 activity assay: Cells were washed twice with ice-cold PBS and sonicated for 30 s in lysis buffer containing 25 mM Tris/0.25 M sucrose (pH 7.4), 0.5 μg/ml leupeptin, 0.5 μg/ml aprotonin, 1 μg/ml pepstatin, and 100 μg/ml phenylmethanesulfonyl fluoride (Sigma Chemical Co.), using a Branson sonifier 250 (VWR Scientific, Willard, OH, USA). The lysates were cooled on ice for 3–5 min and the sonicating-cooling cycle was repeated four more times. Finally, cell lysates were centrifuged at 10,000 × g for 30 min at 4°C and supernatant was used freshly for activity assay. For Western blotting: Enucleated eyes were pooled from age-matched mice of each genotype (WT, ALDH1A1 KO, ALDH3A1 KO *and* DKO), and the corneas were dissected away from the remainder of the eye (10–12 eyes for each genotype). The corneal endothelium was removed by light scraping and the corneal button was homogenized in radioimmunoprecipitation assay buffer in a sonifier for 30s. The lysates were cooled on ice for 3–5 min and the sonicating-cooling cycle was repeated for four more times. The lysate was centrifuged at 10,000×g for 30 min at 4°C and supernatant was collected. Cultured cells were washed in ice-cold PBS and scraped into RIPA buffer (Pierce, Rockford, IL) supplemented with Halt^TM^ protease inhibitor cocktail (Thermo fisher, Waltham, MA) and homogenized and centrifuged for supernatant as noted above for whole cell lysates. Subcellular fractionation on the cultured cells was performed using the NE-PER^TM^ kit (Pierce, Rockford, IL) per the manufacturer’s instructions. The total protein in the supernatant was quantified using the BCA method (Pierce, Rockford, IL).

### Western blot analysis

Ten to 30 μg of protein was subjected to SDS-PAGE and transferred onto a PVDF (Millipore, Billerica, MA) membrane, blocked with 5% non-fat dry milk in TBS-T (0.1% Tween 20) for 2 hr at room temperature. Membranes were incubated overnight at 4°C with primary antibodies against p53 (FL-393, 1:200, Santa Cruz, CA), phosphor-p53-Ser 15 (#9284, 1:1000; Cell signaling Technology, Beverly, MA) or ALDH3A1 (1:2, mouse monoclonal). They were then washed with TBS-T and probed with the appropriate HRP-conjugated secondary antibodies (1:5000, Jackson ImmunoResearch Laboratories, West Grove, PA). Blots were re-probed for β-actin (1:10,000, Sigma, St. Louis, MO), GAPDH (1:300, Santa Cruz, CA) and PARP-1 (1:200, Santa Cruz, CA). β-actin was used as total loading control. GAPDH and PARP-1 were used as cytosolic and nuclear fractionation controls, respectively. Blots were visualized using ECL-plus chemiluminescent substrate (Pierce, Rockford, IL) on the STORM^™^ Imager (GE Life Sciences, Cleveland, Ohio) in blue fluorescence mode. Densitometry on representative images was conducted using ImageQuant TL software (GE Life Sciences, Cleveland, Ohio), where respective protein expression was normalized to their respective loading controls, β-actin, GAPDH and PARP-1. The respective fold changes were then normalized to the protein expression in untreated control samples (wild-type or hTCEpi-TR-lenti). Blots are representative of two independent experiments.

### Statistics

Data are presented as the mean ± standard error (N = 3~6). For BrDU labeling, student’s unpaired t-test was used to test for statistical significance between DKO and WT sections. For cell proliferation study, a two-way ANOVA was used to identify differences between cumulative cell numbers at every passage, with cell line and tetracycline treatment as variables. A Student’s unpaired t-test was used to determine differences between TET-treated and untreated (control) groups for cumulative population doublings and for population doubling time. Evaluation of differences between multiple treatment groups in Q-PCR were conducted using a two-way ANOVA on ΔCt of gene expression, with cell line and tetracycline treatment in low and high calcium conditions included as variables. All pair-wise multiple comparison procedures between means were performed using the Holm-Sidak method. In all experiments, *p* < 0.05 was considered significant.

## Results

### Characterization of Tet-On hTCEpi-ALDH3A1 cell lines

**Fig 1:**
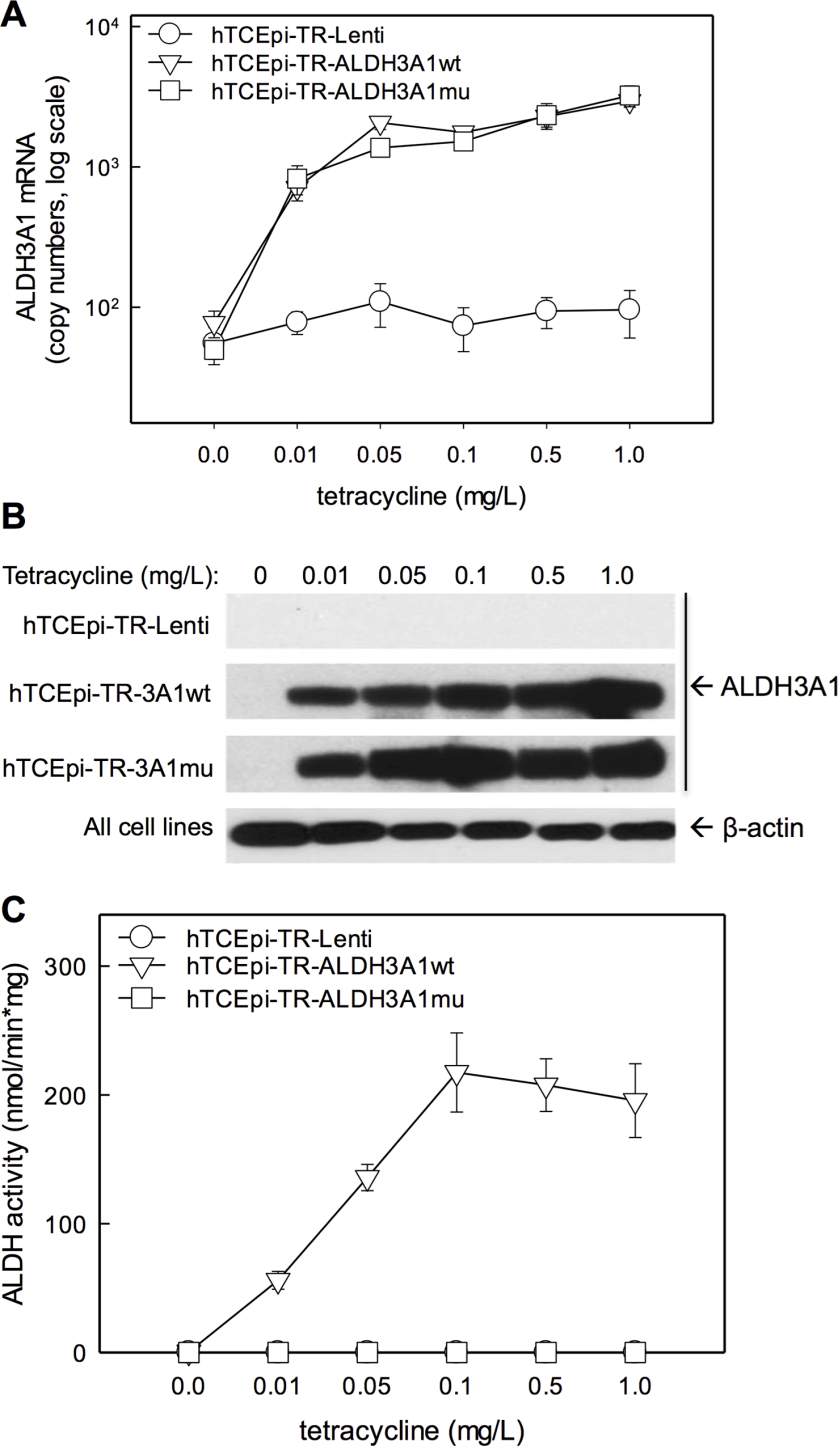
Characterization of Tet-On hTCEpi-ALDH3A1 cell lines. hTCEpi cell lines were transduced with viral particles to stably express wild-type ALDH3A1 (hTCEpi-TR-ALDH3A1wt), mutant (catalytically-inactive) ALDH3A1 (hTCEpi-TR-ALDH3A1mu) or no ALDH3A1 (hTCEpi-TR-Lenti) under the control of a tetracycline-sensitive repressor. Following tetracycline treatment (0–1.0 mg/L) for 72 hr, (**A**) induction of ALDH3A1 mRNA was measured by Q-PCR; mRNA abundance in copy numbers is presented as mean ± standard error (n = 3). (**B**) Induction of ALDH3A1 protein was determined by Western blotting. (**C**) ALDH3A1 catalytic activity was assayed by measuring NADPH production using benzaldehyde as a substrate; data are presented as mean ± standard error (n = 3).

Previously, virally transformed corneal epithelial cell lines have been used as models in studying corneal epithelial biology. However, genetic instability and lack of normal growth and differentiation makes them inadequate to study mechanisms governing corneal homeostasis [20]. We used parent hTCEpi cells, which lack endogenous expression of ALDH3A1 and were shown to stratify, differentiate and desquamate *in vitro* [14]. We established stable cell lines that, upon tetracycline treatment, express either wild-type ALDH3A1 (hTCEpi-TR-ALDH3A1wt) or mutant ALDH3A1 (hTCEpi-TR-ALDH3A1mu). Mutant ALDH3A1 carries a C244A mutation, where the catalytic cysteine residue was replaced with alanine thus abolishing its catalytic activity [18, 19]. To establish a mock-control cell line, we initially developed hTCEpi-TR-LacZ cells, which express bacterial β-galactosidase under the control of tetracycline. For unknown reasons, these cells failed to thrive in culture beyond third passages (data not shown). We therefore used the parental hTCEpi-TR-Lenti cells as the control cell line for our studies. We assayed the transduced cell lines for expression of ALDH3A1 mRNA, protein and catalytic activity. No ALDH3A1 mRNA, protein or catalytic activity was observed at any tetracycline concentration in hTCEpi-TR cells (Fig 1A–1C). In hTCEpi-TR-ALDH3A1wt cells, tetracycline elicited a dose-dependent increase in ALDH3A1 mRNA (Fig 1A), protein (Fig 1B) and activity (Fig 1C). Dose-dependent increases in ALDH3A1mu mRNA (Fig 1A) and protein (Fig 1B) were also induced by tetracycline treatment of hTCEpi-TR-ALDH3A1mu cells, whereas no enzyme activity was detectable in these cells. Since the highest expression of mRNA, protein (ALDH3A1wt or ALDH3A1mu) and activity (which plateaued after 0.1 mg/L TET) were noted at a dose of 1 mg/L TET, all additional experiments involving these transduced cells were performed at this concentration.

### Wild-type and mutant ALDH3A1 expression correlates with decreased corneal epithelial proliferation

**Fig 2:**
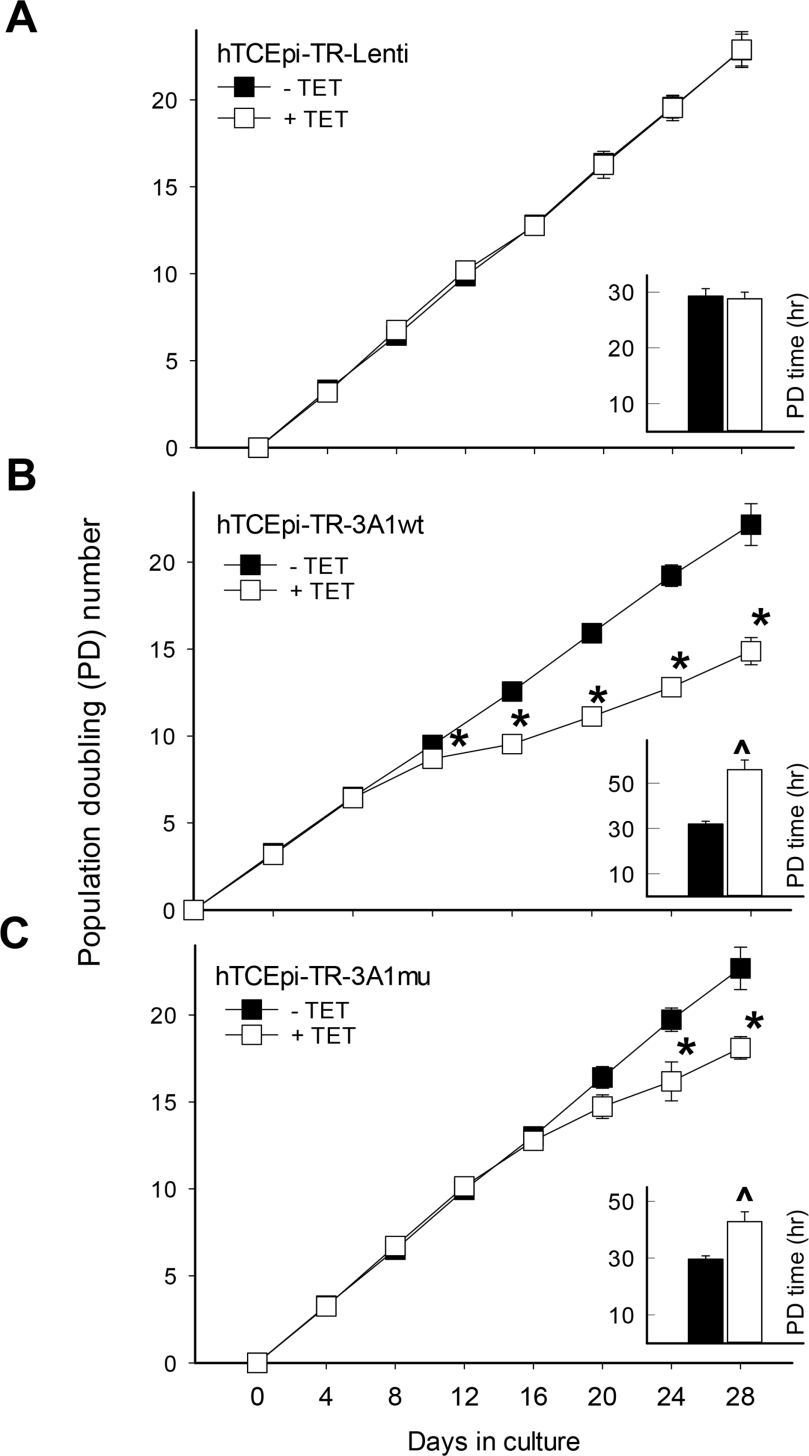
Overexpression of wild-type and mutant ALDH3A1 retards hTCEpi cell proliferation. The effect of ALDH3A1 on cell proliferation was investigated in Tet-On hTCEpi cell lines induced to express (**A**) no ALDH3A1 (hTCEpi-TR-Lenti), (**B**) wild-type ALDH3A1 (hTCEpi-TR-3A1wt) or (**C**) catalytically-inactive mutant ALDH3A1 (hTCEpi-TR-3A1mu). Cells were seeded in 100 mm culture dishes (2.0 x 10^5^ cells/dish) and treated with (+TET) or without (-TET) tetracycline (1μg/ml). After 4 d, cells were trypsinized and viable cells were counted using trypan blue exclusion on a hemocytometer. Consecutively, 2.0 x 10^5^ cells were transferred to a new dish and this process repeated for a total of 8 passages, i.e., for 32 d until treated cells showed decreasing cell numbers. Cumulative population doubling (PD) number at each passage was calculated as: CPD number = ln [N_E_/N_B_] / ln 2 + CPD_o_ (N_E_: end cell count; N_B_: cell count in the beginning; CPD: CPD_o_ at previous passage). Results are reported as mean + standard error from n = 3 experiments. Error bars are contained within the symbols. *p < 0.05, Students unpaired t-test, compared to PD numbers of untreated cells (-TET) at the same passage. ^*p* < 0.05, Students unpaired t-test, compared to the PD time (hr) of untreated cells (-TET).

In order to examine the effect of ALDH3A1 on cell proliferation, transduced cell lines were treated continuously with TET (1 mg/L) to induce sustained ALDH3A1 expression over 8 passages (4-day per passage). TET treatment had no effect on the proliferation rate of hTCEpi-TR-Lenti control cells (Fig 2A). By contrast, TET decreased the proliferation rate of hTCEpi-TR-3A1wt cells beginning at passage 4 (day 12) as reflected by the reduced cumulative population doubling (PD) numbers (Fig 2B). A similar phenomenon was observed for hTCEpi-TR-3A1mu cells except that the growth retardation was observed at a later passage (day 20) (Fig 2C). Overall PD time (hour) was calculated based on the growth curve within this 32-day culturing period (Fig 2A–2C, ***insets***). Compared with hTCEpi-TR-Lenti control cells, the PD times were increased for both hTCEpi-TR-3A1wt and hTCEpi-TR-3A1mu expressing cells by 1.8- and 1.3-fold, respectively. Importantly, the PD times of 3A1wt cells were significantly higher than that of 3A1mu cells (*P* = 0.042).

### p53 expression in hTCEpi-TR cell lines

**Fig 3:**
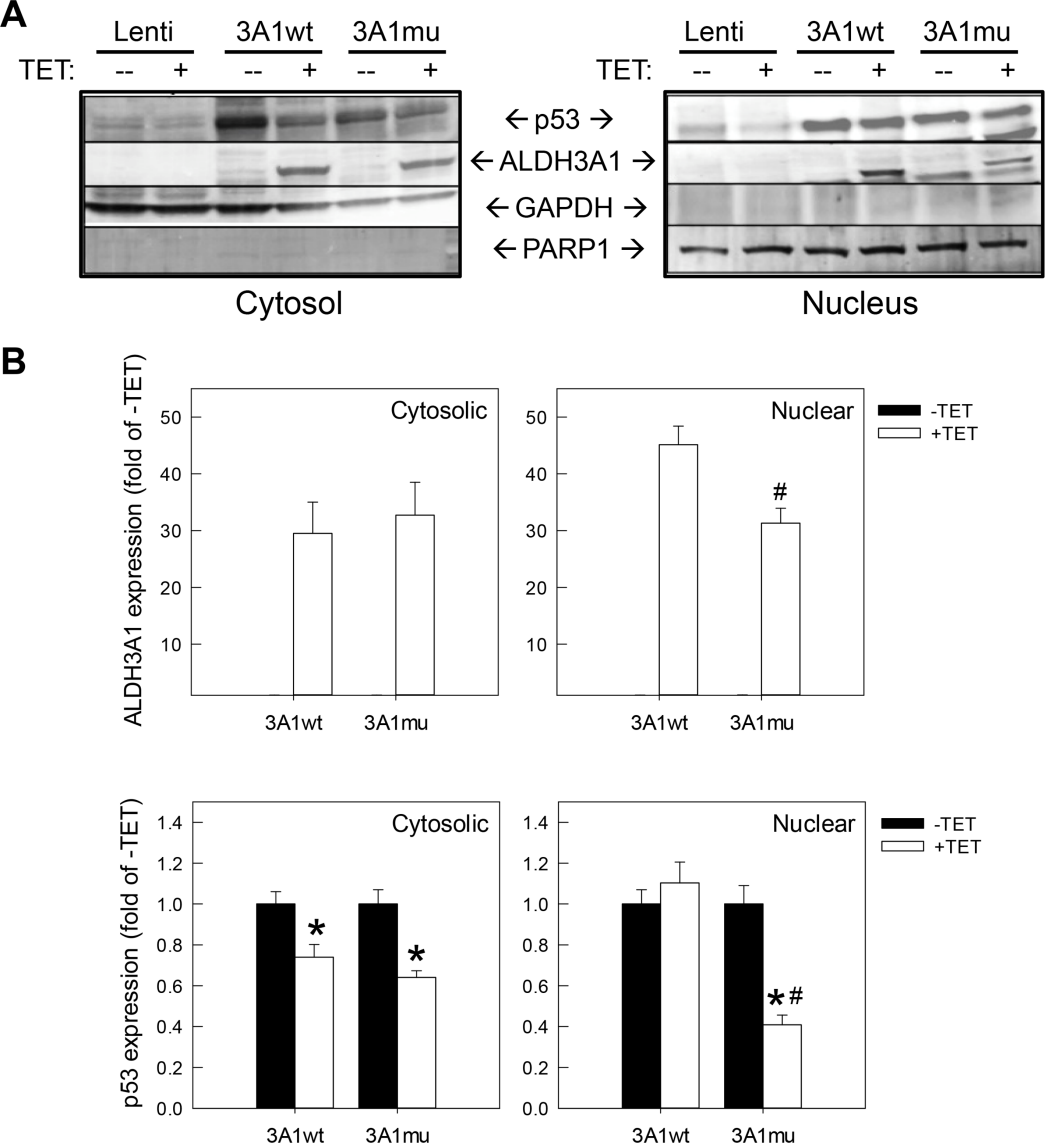
Subcellular levels of p53 protein in hTCEpi-TR cell lines. (**A**) Cytosolic and nuclear fractions from cell lysates of hTCEpi-TR-ALDH3A1wt and hTCEpi-TR-ALDH3A1mu cells treated with vehicle (-TET) or tetracycline (1μg/ml) (+TET) were immunoblotted for p53, ALDH3A1, GAPDH or PARP1 expression. (**B**) Relative levels of ALDH3A1 (upper panels) and p53 (lower panels) expression by densitometry analysis of immunoblots. Results are expressed as the fold of untreated cells (-TET) after correction for respective loading controls (GAPDH for cytosolic extracts; PARP-1 for nuclear extracts). Data are presented as mean + S.E.M (N = 3). *p <0.05, Students unpaired t-test, compared to untreated cells (-TET) of the same cell line. ^#^*p* <0.05, Students unpaired t-test, compared to treated (+TET) ALDH3A1wt cells.

Very high levels of cytoplasmic p53 were noted in corneal epithelia of various vertebrates, including rodents [21, 22]. Cytoplasmic p53 in mouse corneal epithelium was found to be functionally active and responsive to UV-induced DNA damage, exclusively through post-translational stabilization [23]. P53 also plays an indirect role in corneal wound healing [24, 25]. To explore a role of p53 in ALDH3A1 mediated effects, we examined p53 expressions in Tet-On hTCEpi-TR cell lines by Western blotting analysis (Fig 3A & 3B). As expected, TET-induced expression of ALDH3A1 wt and mutant proteins was detected in both cytosolic and nuclear fractions from respective cell lines. TET treatment *per se* did not change p53 expression as seen in hTCEpi-TR-lenti cells. However, endogenous p53 expression (in untreated cells) was much higher in two ALDH3A1-expressing cell lines than in control cells. Interestingly, overexpression of ALDH3A1wt reduced cytosolic, but not nuclear, p53 accumulation, whereas overexpression of ALDH3A1mu decreased p53 levels in both subcellular compartments.

### Dual loss of ALDH1A1 and ALDH3A1 correlates with increased proliferation of corneal epithelium *in vivo*

**Fig 4:**
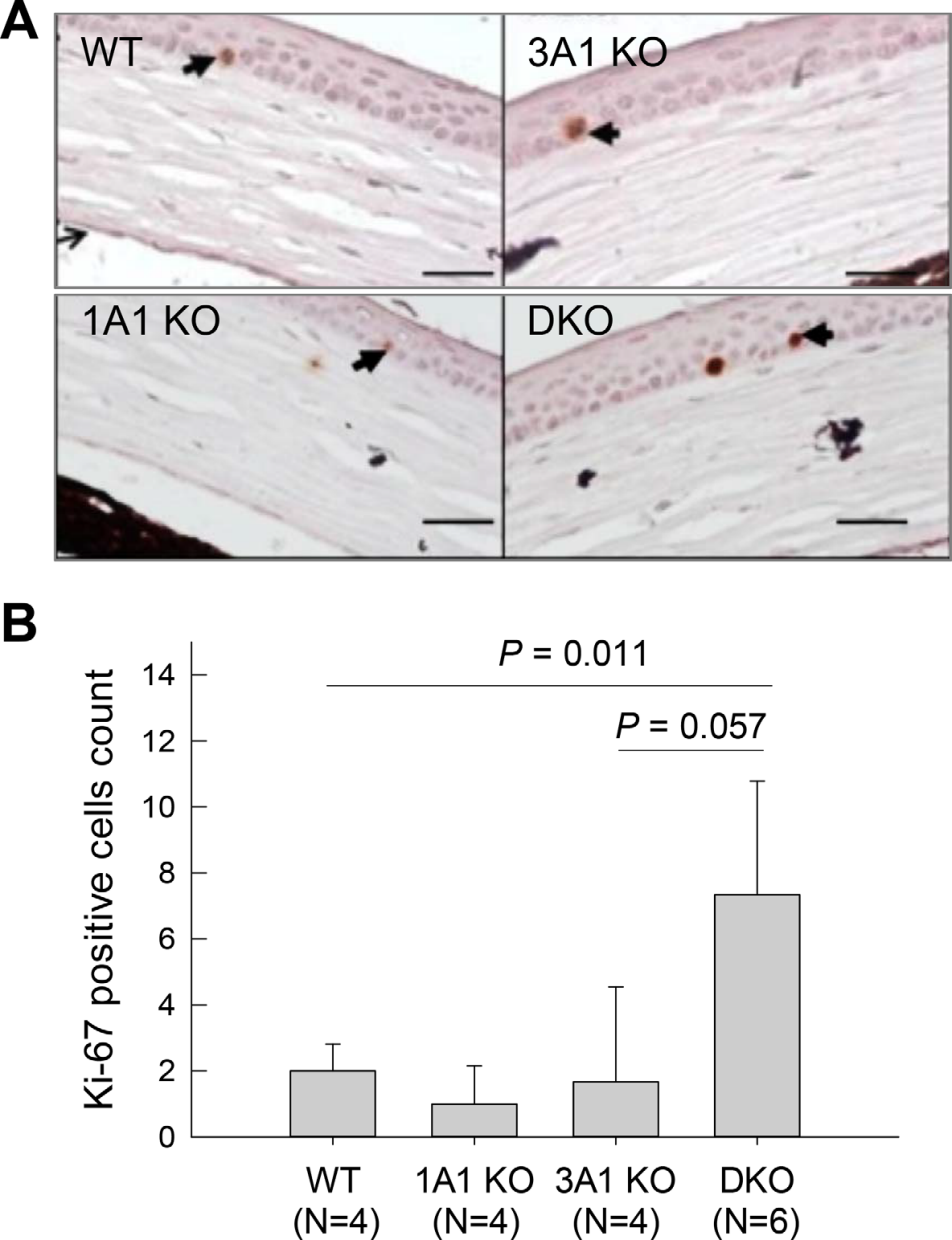
Ki-67 immunohistochemistry of corneal tissues. (**A**) Representative images of Ki-67 IHC of corneal tissues from WT, 1A1 KO, 3A1 KO and DKO mice; scale bar = 50μm. Red staining reflects nuclear staining of Ki-67 (arrows). (**B**) Number of Ki-67-positive nuclei was counted in the central cornea. Data are presented as mean + standard error (N = 4–6). *p < 0.05, Students unpaired t-test, compared to WT.

Ki-67 is a marker for actively cycling cells. Immunohistochemistry (IHC) of corneal epithelium of WT, 1A1 KO, 3A1 KO and DKO mice for this marker showed a significant increase in proliferating corneal epithelial basal cells only in DKO animals (Fig 4A & 4B) relative to the wild-type animals. The differences in Ki-67 positive cell count between 3A1 KO and DKO mice were not significant. However, the difference may have been more significant had more animals been used.

**Fig 5:**
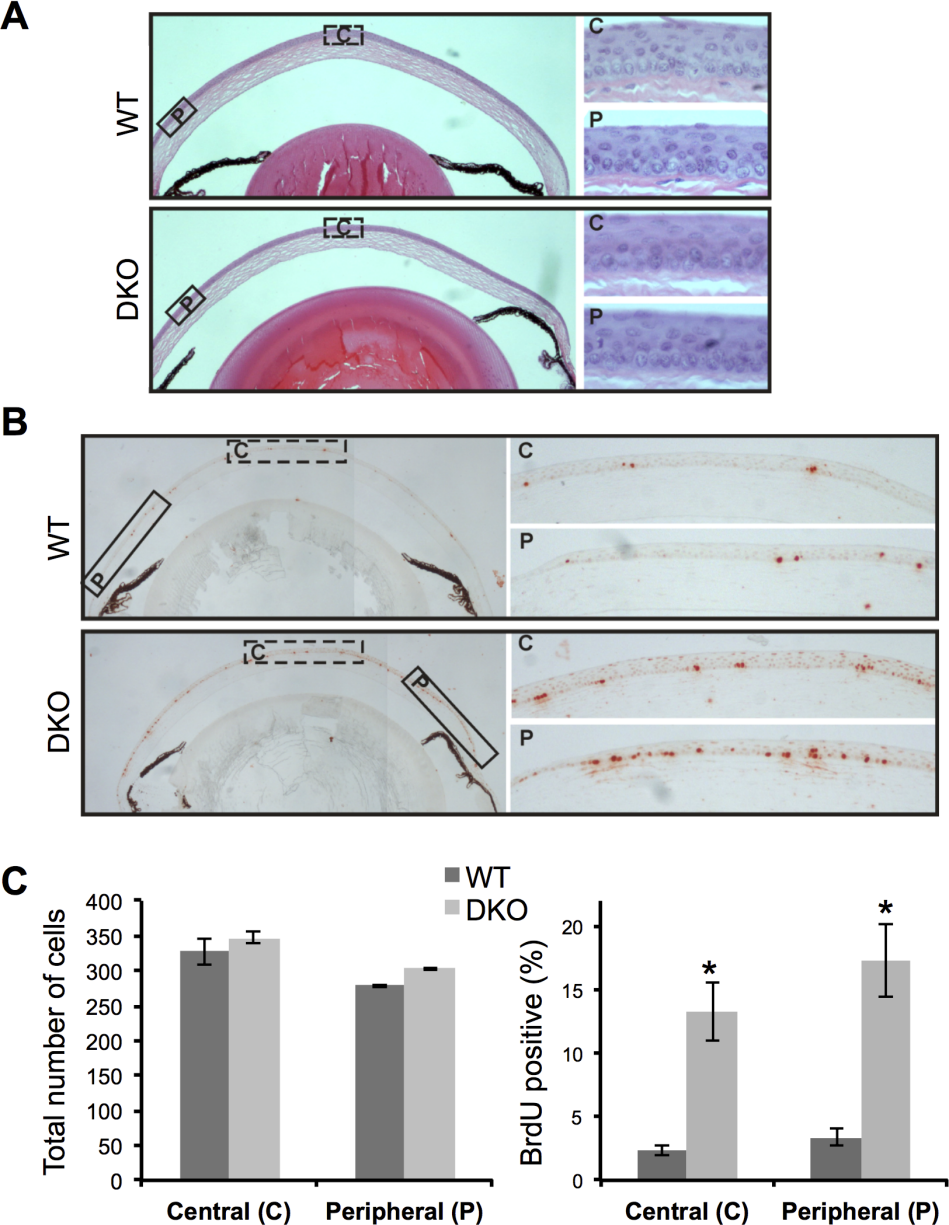
BrDU labeling of corneal epithelial cells. (**A**) Representative H&E staining of corneas from WT and DKO mice; scale bar = 50 μm. Higher magnification images of central (C- dotted box) and peripheral (P-solid box) cells are shown in the right panels; scale bar = 5 μm. (**B**) Representative images of BrDU immunostaining of corneas from WT and DKO mice; scale bar = 100 μm. Higher magnification of central (C) and peripheral (P) corneas are shown in the right panels; scale bar = 25μm. (**C**) The numbers of total cells (left panel) in the central and peripheral regions of corneal epithelium were counted in WT and DKO mice. The numbers of BrDU-positive cells (right graph) in these regions were counted and expressed as a percentage of total cells in the same visual field. Data represent the mean ± standard deviation (N = 3). *p < 0.05, Students unpaired t-test, compared to WT.

Increased proliferation in DKO corneal epithelium was further confirmed by BrDU incorporation staining (Fig 5A–5C), which is indicative of DNA synthesis in proliferating cells. In addition, we investigated the potential differences in proliferation between the central and peripheral epithelium. H&E staining of the cornea revealed no differences in total cell numbers in central or peripheral regions between WT and DKO corneas (Fig 5A). However, corneas from DKO mice had increased levels of BrDU incorporation into the nuclei of the basal layers and suprabasal layers of both central and peripheral epithelial cells, relative to WT mice (Fig 5B & 5C).

### Dual loss of ALDH1A1 and ALDH3A1 leads to loss of p53 expression *in vivo*

**Fig 6:**
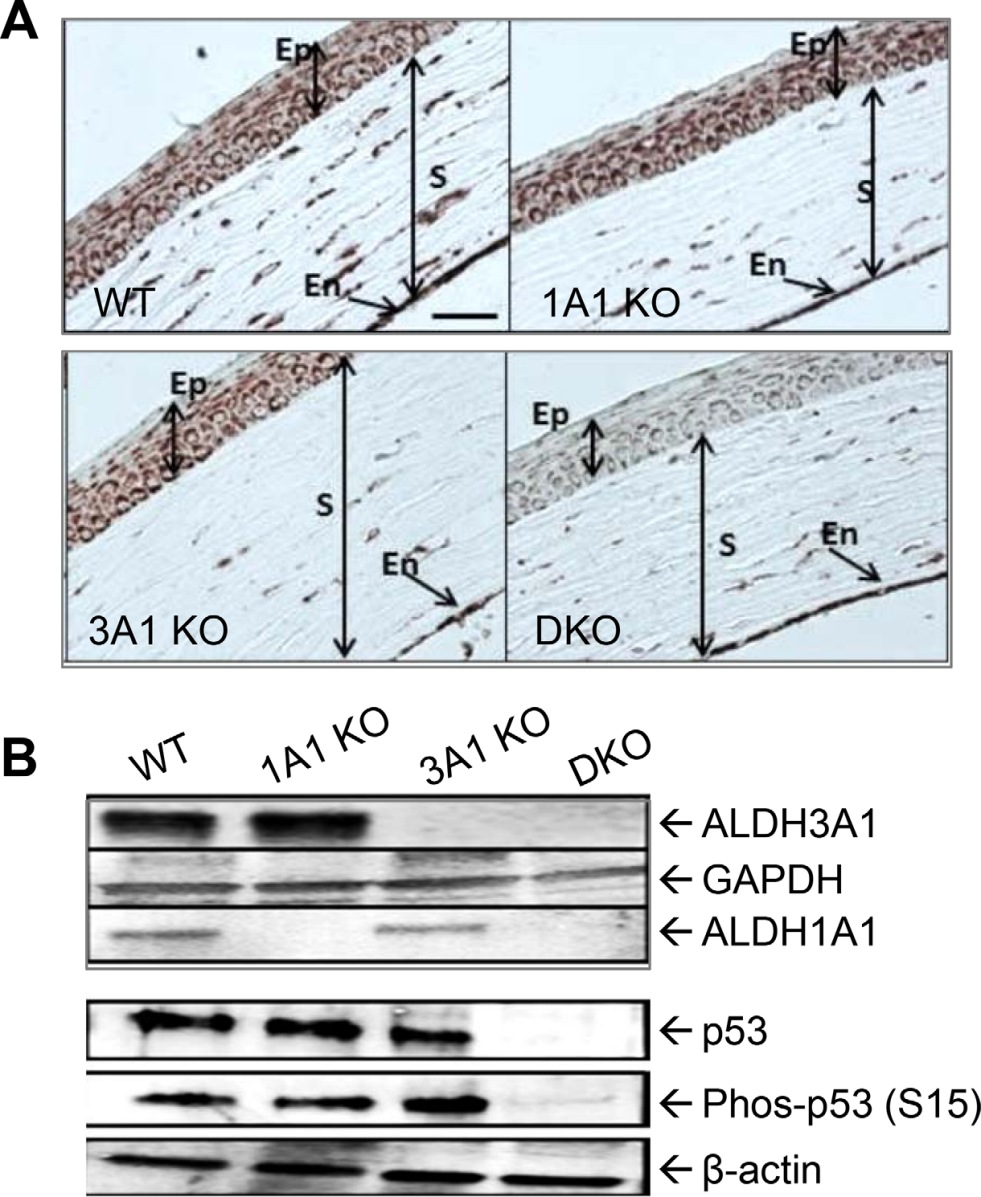
p53 expression in mouse corneas. (**A**) Representative image of IHC staining for p53 in the corneas from WT, 1A1 KO, 3A1 KO and DKO mice; scale bar = 50μm. Cell populations under examination included the epithelium (Ep), stroma (S) and endothelium (En). (**B**) Western blotting for ALDH3A1, ALDH1A1, total p53, and phosphorylated p53 (phos-p53 (S15)) in the mouse corneal whole cell lysates. Amounts of proteins loaded were 2 μg for ALDH3A1, 10 μg for ALDH1A1 and 20 μg for p53 and phos-p53. GAPDH and β-actin were used as loading controls.

IHC analyses of the corneas showed a prominent decrease in p53 positive staining in the epithelial cell layers of DKO mice, although no change was observed in either ALDH1A1 KO or ALDH3A1 KO mice (Fig 6A) compared to the WT controls. The staining was predominantly cytosolic with some perinuclear staining in basal cell layer. Consistent with IHC staining, Western blotting revealed loss of total and phosphorylated p53 proteins in DKO mice (Fig 6B).

### ALDH3A1 expression modifies the expression profile of selective corneal differentiation markers

To explore a potential role of ALDH3A1 in modulating corneal epithelial differentiation, we assayed the mRNA levels of a panel of corneal differentiation markers in Tet-On hTCepi cells undergoing high calcium (1.15 mM) induced differentiation. Cells from all three lines revealed similar morphological changes under high calcium culture condition for one week (data not shown). In high cellular concentration of Ca, mRNA levels of induced ALDH3A1 did not change in hTCEpi-TR-3A1wt cells, but decreased in hTCEpi-TR-3A1mu cells (S1 Table). Additionally, these cells exhibit differential expression patterns of examined marker genes (Table 1).

**Table 1:** Relative abundance of mRNA of corneal differentiation markers.

	**K3**	**K12**	**CX43**	**DSG-2**	**K14**	**IVL**
**A**. -TET (ratio of high Ca/low Ca)						
TR-Lenti	3.1 ± 1.5	2.7 ± 0.4	2.9 ± 0.2	1.4 ± 0.1	2.3 ± 0.1	105 ± 15
TR-3A1wt	2.5 ± 0.7	8.5 ± 1.7 ^a^^,^^b^	1.9 ± 0.3	1.8 ± 0.2	9.2 ± 3.1 ^a^	142 ± 32 ^b^
TR-3A1mu	2.8 ± 0.9	2.5 ± 0.3	2.5 ± 0.4	1.3 ± 0.1	6.3 ± 0.3 ^a^	35 ± 5 ^a^
**B**. Low Ca (ratio of +TET/-TET)						
TR-Lenti	1.1 ± 0.3	0.9 ± 0.2	0.9 ± 0.2	1.1 ± 0.1	1.1 ± 0.1	0.9 ± 0.1
TR-3A1wt	3.9 ± 0.7^a^^,^^b^	2.3 ± 0.8 ^a^^,^^b^	1.0 ± 0.2	1.0 ± 0.1	1.2 ± 0.3	0.2 ± 0.05 ^a^^,^^b^
TR-3A1mu	1.3 ± 0.2	1.1 ± 0.2	1.1 ± 0.3	1.0 ± 0.2	1.3 ± 0.2	1.4 ± 0.2
**C**. High Ca (ratio of +TET/- TET)						
TR-Lenti	0.8 ± 0.1	0.9 ± 0.1	1.3 ± 0.1	1.0 ± 0.1	0.9 ± 0.1	0.8 ± 0.1
TR-3A1wt	52 ± 15 ^a^	95 ± 31 ^a^^,^^b^	2.0 ± 0.4	1.3 ± 0.1	0.15 ± 0.03 ^a^	0.13 ± 0.03 ^a^
TR-3A1mu	29 ± 9 ^a^	31 ± 12 ^a^	1.5 ± 0.2	1.1 ± 0.2	0.13 ± 0.02 ^a^	0.2 ± 0.05 ^a^

mRNA expression was measured by Q-PCR in hTCEpi cell lines that express no ALDH3A1 (TR-Lenti) or wild-type ALDH3A1 (TR-3A1wt) or inactive mutant ALDH3A1 (TR-3A1mu) induced by tetracycline (TET) treatment (1 mg/L) for 7 days. During this period, cells were incubated with medium containing either 0.15 mM calcium (Low Ca) or 1.15 mM Calcium (High Ca). The relative mRNA abundance is reported as the ratio of expression between conditions as specified after normalization with housekeeping gene GAPDH. Data represent mean ± standard error (N = 3). Under the same category, by Student’s unpaired *t*-test with Holm-Sidak adjustment

^a^: *P* < 0.05, when compared with TR-Lenti cells

^b^: *P* < 0.05, when compared with TR-3A1mu cells. K3, keratin 3

K12, keratin 12; CX43, connexin43; DSG-2, hemi desmosome protein desmoglein 2; K14, keratin 14; IVL, involucrin.

Keratin expression is often associated with differentiation in epithelial cells and is primarily controlled at the level of transcription. Keratin 3 (K3) and keratin 12 (K12) are differentiation markers specific for corneal epithelial cells. High calcium (Table 1, A) alone induced a ~3-fold induction of K3 mRNA in all three cell types. In low calcium culture conditions (Table 1B), TET treatment induced a 4-fold increase in K3 mRNA in hTCEpi-TR-3A1wt cells only. No such induction occurred in hTCEpi-TR-Lenti or hTCEpi-TR-3A1mu cells. Upon TET treatment in high calcium conditions (Table 1C), wild-type and mutant cells both showed a dramatic increase in K3 mRNA expressions (50- and 30-fold) over their respective control treatments. Similar pattern was observed for the K12 transcript. High calcium alone (Table 1A) caused a much higher induction (8-fold) in hTCEpi-TR-3A1wt cells relative to that in control and hTCEpi-TR-3A1mu cells (2-fold). Under low calcium conditions (Table 1B), TET treatment caused a 2-fold increase in K12 mRNA only in hTCEpi-TR-3A1wt cells and had no such effect in either control or mutant cells. By contrast, under high calcium conditions (Table 1C), TET treatment induced a drastic increase in K12 mRNA levels in hTCEpi-TR-3A1wt (80-fold) and to a lesser extend in hTCEpi-TR-3A1mu cells (20- fold).

Connexin 43 (Cx43) and desmoglein 2 (DSG2) respectively are late and early differentiation markers for corneal epithelium. High calcium conditions (Table 1A) alone induced a 2- to 3- fold induction of Cx43 mRNA in all three cell lines. Under low calcium conditions (Table 1B), TET treatment had no effect on Cx43 mRNA expression in any of these cells; whereas under high calcium conditions (Table 1C), TET treatment increased Cx43 mRNA mildly (1.3- to 2-fold) in all of the cell lines. This is likely due to the primary effect of high calcium, not because of ALDH3A1 expression. For DSG2, neither high calcium or TET treatment conditions caused any change in DSG2 mRNA expression in any of the three cell lines.

Keratin 14 (K14) is a squamous epithelial proliferative marker, expressed in basal corneal epithelial cells. Under high calcium condition alone (Table 1A) K14 mRNA was induced 2-fold in hTCEpi-TR-Lenti cells, but much higher in hTCEpi-TR-3A1wt (10-fold) and hTCEpi-TR-3A1mu (6-fold) cells. Under low calcium conditions (Table 1B), TET treatment had no effect on K14 mRNA in any cell lines. However, under high calcium conditions (Table 1C), TET treatment drastically decreased K14 mRNA expressions in both hTCEpi-TR-3A1wt and hTCEpi-TR-3A1mu cells, indicating that ALDH3A1 protein overexpression irrespective of activity may have mediated this effect. Involucrin (IVL) is a terminal differentiation marker, expressed in the most superficial stratified epithelia of the skin and cornea cells. It provides mechanical support and protection against pathogen invasion and mechanical shear stress. In this study, high calcium condition alone (Table 1A) increased greatly the IVL mRNA levels in all three cell lines. The magnitude of the increase was less in hTCEpi-TR-3A1mu cells (35-fold) compared to hTCEpi-TR-3A1wt or hTCEpi-TR-Lenti cells (> 100-fold). In low calcium conditions (Table 1B), TET treatment decreased IVL mRNA expression only in hTCEpi-TR-3A1wt cells, suggesting ALDH3A1 activity may be required in this effect. Under high calcium conditions (Table 1C), TET treatment decreased IVL mRNA in hTCEpi-TR-3A1wt and hTCEpi-TR-3A1mu cells to a comparable degree, suggesting ALDH3A1 protein and not just activity is functional in down-regulating IVL expression.

## Discussion

The mammalian cornea draws a close parallel with their abundant and taxon-specific expression of aldehyde dehydrogenases or “corneal crystallins”. ALDH3A1 has been shown to directly absorb UV light, protect against lipid peroxidation, replenish antioxidant NADPH, scavenge reactive oxygen species and possess chaperone-like activity. The proven enzymatic and non-enzymatic nature of this enzyme and its high concentration in mammalian corneal epithelium supports the idea that gene sharing has allowed corneal ALDHs to take on multiple roles in maintaining corneal function. In this study, we report additional evidence supporting a novel regulatory role for ALDH3A1 in the maintenance of corneal epithelial homeostasis, through modulating cell proliferation and differentiation programs. One line of evidence supporting the hypothesis that ALDH3A1 plays a role in controlling cell proliferation can be found in the developing mouse. During active proliferation in the cornea at post-natal day 9, ALDH3A1 expression is non-existent. At post-natal day 14 (just before the eyelid opening), expression of ALDH3A1 is drastically up-regulated; this is a time when corneal cell proliferation is reduced [26] and the cornea has reached normal thickness [27].

Our data showing increased BrDU and Ki-67 labeling in *Aldh1a1*^*-/-*^*/3a1*^*-/-*^ DKO mice, especially in basal layers of the epithelium, the site of corneal epithelial proliferation further supports the negative modulation of cell growth by ALDHs. This is surprising since it was expected that *Aldh3a1*^*-/-*^ KO mice would have the highest proliferation, especially given that in wild-type mice the relative expression of ALDH3A1 is higher than that of ALDH1A1. That only simultaneous loss of both ALDHs contributes to increased proliferation suggests that these ALDHs may compensate for each other. Accordingly, we propose that both corneal ALDHs are required as a check against excessive proliferation, which in pathological corneas was shown to be detrimental to transparency.

Our prior investigation suggesting a regulatory role for ALDH3A1 was conducted in immortalized corneal epithelial cells (HCE). These cells however, are known to have altered cell cycle control pathways as a result of SV-40 large T antigen-induced genomic aberrations and heterogeneity [20]. hTCEpi cells have normal cell cycle control and hence chosen as a better model to delineate the mechanism underlying ALDH3A1-induced growth retardation. Through the use of newly developed Tet-On inducible hTCEpi cell lines, we show that induction of ALDH3A1 in these cells led to decreased cell proliferation and, for the first time, we demonstrate that this regulatory effect ascribes partially to the catalytic activity of the enzyme. One likely explanation is that ALDH3A1 may also function through protein-protein interactions with cell cycle modulators.

The regulatory protein p53, often referred to as the ‘guardian of the genome’, is a transcription factor promoting cell cycle arrest, DNA damage repair and apoptosis. Under conditions of stress, p53 is stabilized and accumulates in the nucleus primarily due to post-translational modification (e.g. phosphorylation) [28–30]. High p53 levels have been demonstrated in the cytosol of corneal and conjunctival epithelium of normal cornea of several species [21, 22]. Although previously thought to be inactive [22], a recent study in mouse primary corneal epithelial cells showed that cytosolic p53 can be phosphorylated to functionally induce apoptosis in response to UV irradiation [23]. In these studies, no change in p53 transcription was noted, suggesting that p53 localization in the nucleus was the result of rapid transit of cytoplasmic p53 into the nucleus [28]. In hTCEpi cells overexpressing wild-type ALDH3A1, p53 was decreased in the cytosol, but was stabilized in the nucleus. Fully functioning of this effect requires the catalytic activity of ALDH3A1, as indicated by the observation that p53 was decreased in both the cytoplasm and the nucleus in cells overexpressing mutant ALDH3A1. On the other hand, p53 and phospho-p53 expression was nearly absent in the corneas from mice deficient in both ALDH1A1 and ALDH3A1, but was preserved in the corneas from mice deficient in either ALDH isozyme. Taken together, our results suggest that both ALDH1A1 and ALDH3A1 may be modulating p53 expression *in vivo* in the cornea through mutual compensation and p53 might be involved in the anti-proliferation effects of ALDH3A1 and ALDH1A1.

Cellular proliferation and differentiation are two key elements in the maintenance of corneal epithelium homeostasis. Previous and this current study clearly show that ALDH3A1 plays an inhibitory role in epithelial proliferation. To date, little is known about ALDH3A1-mediated effects on corneal epithelial differentiation. As mentioned earlier, a PAX6 binding site has been identified in the *Aldh3a1* promoter sequence in the mouse cornea [27]. In heterozygous small eye *Pax6(+/-)* mice, decreased PAX6 results in a reduction in ALDH3A1 mRNA levels in the cornea [27]. Reduced PAX6 also correlates with specific defects in actin, Keratin 12 and desmoplakin localization, cell junctional complexes and causes increased oxidative stress [31]. This hints at the possibility of ALDH3A1 serving as a signal transduction molecule for PAX6 in regulating differentiation programs.

In the present study, we adopted a high calcium-induced differentiation model using ALDH3A1 Tet-On hTCEpi cells and observed a significant effect of ALDH3A1 on the expression profile of various corneal epithelial differentiation markers. Specifically, two corneal epithelial specific markers, namely K3 and K12, were markedly up-regulated upon ALDH3A1 expression under high calcium culturing condition and the enzymatic activity of ALDH3A1 likely contributes partially to this effect. It should be noted that, in parent hTCEpi cells, airlifting is required to induce stratification and K3/K12 protein expression [14]. Thus, our results indicate that induced ALDH3A1 expression may act downstream of stratification and upstream of induced K3/K12 expression. Two cell-cell junction proteins CX43 and DSG2 were not significantly altered by ALDH3A1 expression regardless of its enzyme activity or differentiating status. In contrast, two markers of epidermal differentiation, namely K14 and IVL, were induced by high calcium alone in hTCEpi cells, but were greatly suppressed by ALDH3A1 expression, irrelevant to its enzymatic capacity. Taken together, this result is suggestive of a potential role of ALDH3A1 in contributing to cellular commitment to corneal epithelium and away from epidermal differentiation. The molecular details of this ALDH3A1-mediated effect warrant future studies.

**Fig 7:**
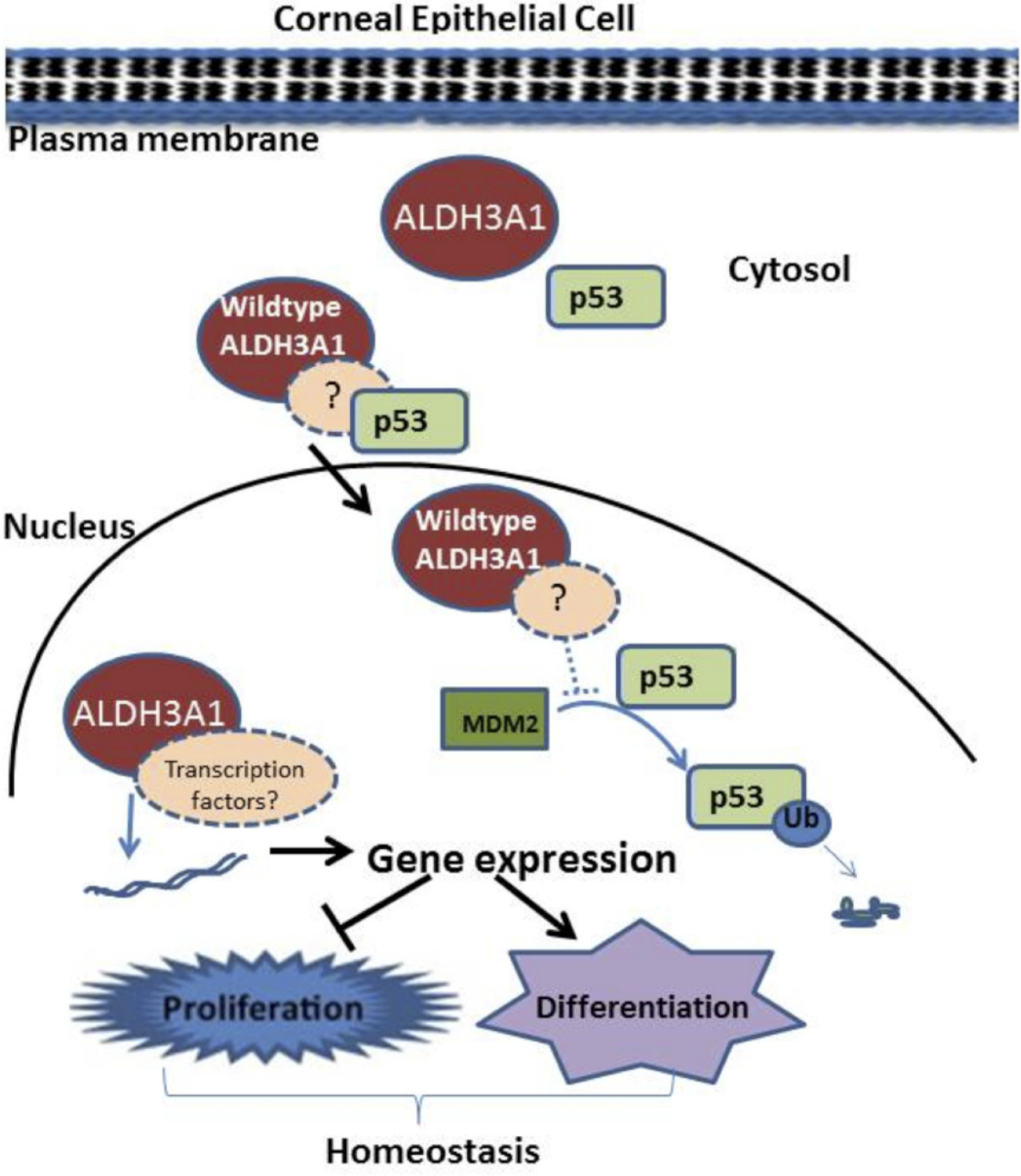
Scheme of proposed regulatory function of ALDH3A1 in corneal epithelial cells. ALDH3A1 in corneal epithelial cells retards proliferation by transcriptional regulation of cell cycle modulators. Wild-type ALDH3A1 contributes to p53 sequestration into the nucleus, while protecting it from degradation. ALDH3A1 protein also contributes to differentiation through association with transcription factors involved in regulating mRNA levels of differentiation specific markers.

In summary, this study provides evidence supporting a novel regulatory role for ALDH3A1 in corneal homeostasis by modulating proliferative and differentiation programs. Mechanistically, ALDH3A1 expression may target expression of specific genes involved in anti-proliferation and pro-differentiation (Fig 7). Many of these actions of ALDH3A1 appear to occur, in part, dependently of its catalytic activity. As yet, protein-protein interactions for ALDH3A1 have not been reported, although such interactions are predicted for other members of the ALDH superfamily, e.g. ALDH16A1 and ALDH1A1 through coiled-coil domains [32, 33] in the protein structure. It would therefore be interesting to investigate ALDH3A1–protein interactions in corneal epithelial cells. It is anticipated that the insights gained about the novel role of ALDH3A1 in regulating corneal epithelial homeostasis may ultimately translate into the development of more effective therapeutic interventions for corneal pathologies, such as dry eye, injury or infection-based corneal scarring and haze.

## Supporting Information

S1 Table Primer sequences used in Q-PCR analysis. (DOCX)

## References

[1] Wilson SE, Netto M, Ambrósio R Jr. Corneal cells: chatty in development, homeostasis,wound healing, and disease. American Journal of Ophthalmology. 2003;136(3):530–6. 10.1016/s0002-9394(03)00085-0 12967809

[2] Piatigorsky J. Enigma of the abundant water-soluble cytoplasmic proteins of the cornea: the "refracton" hypothesis. Cornea. 2001;20(8):853–8. . 11685065 10.1097/00003226-200111000-00015

[3] Piatigorsky J. Lens and cornea: the "refracton hypothesis". Seminars in cell & developmental biology. 2008;19(2):69–70. 10.1016/j.semcdb.2007.10.010 . 18054835

[4] Piatigorsky J. Gene sharing in lens and cornea: facts and implications. Progress in retinal and eye research. 1998;17(2):145–74. . 9695791 10.1016/s1350-9462(97)00004-9

[5] Piatigorsky J. Review: A case for corneal crystallins. J Ocul Pharmacol Ther. 2000;16(2):173–80. Epub 2000/05/10. . 10803428 10.1089/jop.2000.16.173

[6] Chen Y, Thompson DC, Koppaka V, Jester JV, Vasiliou V. Ocular aldehyde dehydrogenases: protection against ultraviolet damage and maintenance of transparency for vision. Progress in retinal and eye research. 2013;33:28–39. Epub 2012/10/27. 10.1016/j.preteyeres.2012.10.001 S1350-9462(12)00071-7 [pii]. 23098688 PMC3570594

[7] Stagos D, Chen Y, Cantore M, Jester JV, Vasiliou V. Corneal aldehyde dehydrogenases: multiple functions and novel nuclear localization. Brain Res Bull. 2010;81(2–3):211–8. Epub 2009/09/02. S0361-9230(09)00265-2 [pii] 10.1016/j.brainresbull.2009.08.017 19720116 PMC3025408

[8] Pappa A, Chen C, Koutalos Y, Townsend AJ, Vasiliou V. Aldh3a1 protects human corneal epithelial cells from ultraviolet- and 4-hydroxy-2-nonenal-induced oxidative damage. Free Radic Biol Med. 2003;34(9):1178–89. Epub 2003/04/23. S0891584903000704 [pii]. . 12706498 10.1016/s0891-5849(03)00070-4

[9] Vasiliou V, Thompson DC, Smith C, Fujita M, Chen Y. Aldehyde dehydrogenases: from eye crystallins to metabolic disease and cancer stem cells. Chem Biol Interact. 2013;202(1–3):2–10. Epub 2012/11/20. 10.1016/j.cbi.2012.10.026 S0009-2797(12)00233-5 [pii]. . 23159885 PMC4128326

[10] Estey T, Cantore M, Weston PA, Carpenter JF, Petrash JM, Vasiliou V. Mechanisms involved in the protection of UV-induced protein inactivation by the corneal crystallin ALDH3A1. J Biol Chem. 2007;282(7):4382–92. Epub 2006/12/13. M607546200 [pii] 10.1074/jbc.M607546200 . 17158879

[11] Pappa A, Brown D, Koutalos Y, DeGregori J, White C, Vasiliou V. Human aldehyde dehydrogenase 3A1 inhibits proliferation and promotes survival of human corneal epithelial cells. J Biol Chem. 2005;280(30):27998–8006. Epub 2005/05/21. M503698200 [pii] 10.1074/jbc.M503698200 . 15905174

[12] Pappa A, Estey T, Manzer R, Brown D, Vasiliou V. Human aldehyde dehydrogenase 3A1 (ALDH3A1): biochemical characterization and immunohistochemical localization in the cornea. Biochem J. 2003;376(Pt 3):615–23. Epub 2003/08/29. 10.1042/BJ20030810 BJ20030810 [pii]. 12943535 PMC1223798

[13] Feng Y, Zhu X, Dang Y, Ma Q. Alkali burn causes aldehyde dehydrogenase 3A1 (ALDH3A1) decrease in mouse cornea. Mol Vis. 2004;10:845–50. Epub 2004/11/18. v10/a101 [pii]. . 15547490

[14] Robertson DM, Li L, Fisher S, Pearce VP, Shay JW, Wright WE, et al Characterization of growth and differentiation in a telomerase-immortalized human corneal epithelial cell line. Invest Ophthalmol Vis Sci. 2005;46(2):470–8. Epub 2005/01/27. 46/2/470 [pii] 10.1167/iovs.04-0528 . 15671271

[15] Nees DW, Wawrousek EF, Robison WG Jr., Piatigorsky J. Structurally normal corneas in aldehyde dehydrogenase 3a1-deficient mice. Mol Cell Biol. 2002;22(3):849–55. Epub 2002/01/11. 11784860 10.1128/MCB.22.3.849-855.2002 PMC133561

[16] Lassen N, Bateman JB, Estey T, Kuszak JR, Nees DW, Piatigorsky J, et al Multiple and additive functions of ALDH3A1 and ALDH1A1: cataract phenotype and ocular oxidative damage in Aldh3a1(-/-)/Aldh1a1(-/-) knock-out mice. J Biol Chem. 2007;282(35):25668–76. Epub 2007/06/15. M702076200 [pii] 10.1074/jbc.M702076200 17567582 PMC2253645

[17] Fan X, Molotkov A, Manabe S, Donmoyer CM, Deltour L, Foglio MH, et al Targeted disruption of Aldh1a1 (Raldh1) provides evidence for a complex mechanism of retinoic acid synthesis in the developing retina. Mol Cell Biol. 2003;23(13):4637–48. Epub 2003/06/17. 12808103 10.1128/MCB.23.13.4637-4648.2003 PMC164835

[18] Luo Y, Dallaglio K, Chen Y, Robinson WA, Robinson SE, McCarter MD, et al ALDH1A isozymes are markers of human melanoma stem cells and potential therapeutic targets. Stem cells. 2012;30(10):2100–13. 10.1002/stem.1193 22887839 PMC3448863

[19] Schmittgen TD, Livak KJ. Analyzing real-time PCR data by the comparative C(T) method. Nat Protoc. 2008;3(6):1101–8. Epub 2008/06/13. . 18546601 10.1038/nprot.2008.73

[20] Yamasaki K, Kawasaki S, Young RD, Fukuoka H, Tanioka H, Nakatsukasa M, et al Genomic aberrations and cellular heterogeneity in SV40-immortalized human corneal epithelial cells. Invest Ophthalmol Vis Sci. 2009;50(2):604–13. Epub 2008/10/01. 10.1167/iovs.08-2239 iovs.08-2239 [pii]. . 18824731

[21] Pokroy R, Tendler Y, Pollack A, Zinder O, Weisinger G. p53 expression in the normal murine eye. Invest Ophthalmol Vis Sci. 2002;43(6):1736–41. Epub 2002/05/31. . 12036973

[22] Tendler Y, Panshin A, Weisinger G, Zinder O. Identification of cytoplasmic p53 protein in corneal epithelium of vertebrates. Experimental Eye Research. 2006;82(4):674–81. 10.1016/j.exer.2005.09.005 16376331

[23] Tendler Y, Pokroy R, Panshin A, Weisinger G. p53 protein subcellular localization and apoptosis in rodent corneal epithelium cell culture following ultraviolet irradiation. International journal of molecular medicine. 2013;31(3):540–6. 10.3892/ijmm.2013.1247 . 23338225

[24] Feng Z, Hu W, de Stanchina E, Teresky AK, Jin S, Lowe S, et al The Regulation of AMPK β1, TSC2, and PTEN Expression by p53: Stress, Cell and Tissue Specificity, and the Role of These Gene Products in Modulating the IGF-1-AKT-mTOR Pathways. Cancer Research. 2007;67(7):3043–53. 10.1158/0008-5472.can-06-4149 17409411

[25] Wang Y, Zhao X, Shi D, Chen P, Yu Y, Yang L, et al Overexpression of SIRT1 Promotes High Glucose–Attenuated Corneal Epithelial Wound Healing via p53 Regulation of the IGFBP3/IGF-1R/AKT Pathway. Investigative Ophthalmology & Visual Science. 2013;54(5):3806–14. 10.1167/iovs.13-12091 23661372

[26] Zieske JD. Corneal development associated with eyelid opening. Int J Dev Biol. 2004;48(8–9):903–11. Epub 2004/11/24. 041860jz [pii] 10.1387/ijdb.041860jz . 15558481

[27] Davis J, Davis D, Norman B, Piatigorsky J. Gene expression of the mouse corneal crystallin Aldh3a1: activation by Pax6, Oct1, and p300. Invest Ophthalmol Vis Sci. 2008;49(5):1814–26. Epub 2008/04/26. 49/5/1814 [pii] 10.1167/iovs.07-1057 . 18436815

[28] Maxwell SA, Roth JA. Posttranslational regulation of p53 tumor suppressor protein function. Crit Rev Oncog. 1994;5(1):23–57. Epub 1994/01/01. . 7948107 10.1615/critrevoncog.v5.i1.20

[29] Ryan KM, Phillips AC, Vousden KH. Regulation and function of the p53 tumor suppressor protein. Curr Opin Cell Biol. 2001;13(3):332–7. Epub 2001/05/10. S0955-0674(00)00216-7 [pii]. . 11343904 10.1016/s0955-0674(00)00216-7

[30] Efeyan A, Serrano M. p53: guardian of the genome and policeman of the oncogenes. Cell Cycle. 2007;6(9):1006–10. Epub 2007/04/26. 4211 [pii]. . 17457049 10.4161/cc.6.9.4211

[31] Ou J, Lowes C, Collinson JM. Cytoskeletal and Cell Adhesion Defects in Wounded and Pax6+/− Corneal Epithelia. Investigative Ophthalmology & Visual Science. 2010;51(3):1415–23. 10.1167/iovs.09-4023 19933176

[32] Vasiliou V, Sandoval M, Backos DS, Jackson BC, Chen Y, Reigan P, et al ALDH16A1 is a novel non-catalytic enzyme that may be involved in the etiology of gout via protein-protein interactions with HPRT1. Chem Biol Interact. 2013;202(1–3):22–31. Epub 2013/01/26. 10.1016/j.cbi.2012.12.018 S0009-2797(13)00006-9 [pii]. 23348497 PMC3746320

[33] Chen Y, Koppaka V, Thompson DC, Vasiliou V. Focus on molecules: ALDH1A1: from lens and corneal crystallin to stem cell marker. Exp Eye Res. 2012;102:105–6. Epub 2011/05/04. 10.1016/j.exer.2011.04.008 S0014-4835(11)00130-8 [pii]. 21536030 PMC3423494

