# Supplementary material for: ALDH3A1 Plays a Functional Role in Maintenance of Corneal Epithelial Homeostasis
Source: PLoS One. 2016 Jan 11;11(1):e0146433. doi: 10.1371/journal.pone.0146433 (PMC4708999; doi:10.1371/journal.pone.0146433)
Supplement: S1 Table — (DOCX) [file pone.0146433.s001.docx]

**Supporting Information**

**S1 Table.** **Primer sequences used in Q-PCR analysis**

| **Product** | **Forward** | **Reverse** |
| --- | --- | --- |
| Keratin 3 | GTCCTGGAGACCAAGTGGAA | CACCAGGTCCTCCATGTTCT |
| Keratin 12 | TTCTGCTGCTTCCATGTTTG | TCATTGCCCGAGAGAATACC |
| Connexin 43 | AGCAGTTGAGTAGGCTTGAAC | ACTTGGCGTGACTTCACTAC |
| Desmoglein-2 | GCCATCTCTTGCTTCTACTGTC | GCTTATCCTCCAGTGTTCTACC |
| Keratin 14 | TTCTGAACGAGATGCGTGAC | GCAGCTCAATCTCCAGGTTC |
| Involucrin | GATGTCCCAGCAACACACAC | TGCTCACATTCTTGCTCAGG |
| GAPDH | GCAACAATATCCACTTTACCAGAG | CACATCGCTCAGACACCAT |
